# Family Loneliness: Its Effects in the Development of Empathy, Teamwork and Lifelong Learning Abilities in Medical Students

**DOI:** 10.3389/fpsyg.2020.02046

**Published:** 2020-08-18

**Authors:** Nancy Berduzco-Torres, Pamela Medina, Begonia Choquenaira-Callañaupa, Montserrat San-Martín, Roberto C. Delgado Bolton, Luis Vivanco

**Affiliations:** ^1^Escuela Profesional de Enfermería, Universidad Nacional San Antonio Abad del Cusco, Cusco, Peru; ^2^Departamento de Estadística e Investigación Operativa, Universidad de Granada, Melilla, Spain; ^3^Servicio de Medicina Nuclear, Centro de Investigación Biomédica de La Rioja (CIBIR), Logroño, Spain; ^4^Plataforma de Bioética y Educación Médica, Centro de Investigación Biomédica de La Rioja (CIBIR), Logroño, Spain; ^5^Centro Nacional de Documentación en Bioética, Fundacion Rioja Salud, Logroño, Spain; ^6^Area de Salud, Nutrición y Bioética, Fundación Universitaria Iberoamericana (FUNIBER), Barcelona, Spain

**Keywords:** attachment relationship, intergenerational solidarity theory, empathy, teamwork, lifelong learning, loneliness, subjective well-being, medical students

## Abstract

**Context:**

Family offers an important source of social support where individuals acquire social abilities that are necessary to create positive human relationships. This influence has been discussed by different sociological and psychological theories along the life span of individuals. In medicine, empathy, teamwork, and lifelong learning have been described as specific elements of professionalism that have special importance in the interaction with patients and in physicians’ well-being at the workplace. This study was performed with the aim of demonstrating the following hypothesis: In the absence of specific training in empathy and teamwork and lifelong learning abilities, their development in medical students is associated with the students’ perception of loneliness from their family environment.

**Methods:**

A cross-sectional study was performed in the only two medical schools of Cusco (Peru), one private and the other public. Jefferson Scales of Empathy, Teamwork, and Lifelong Learning were used as the main measures. Mother–son and father–son relationships and family loneliness were measured to characterize the family environment. In addition, information related to sex, medical school, academic achievements, and place of origin were collected to control possible biases. Comparative, correlation, and multiple regression analyses were performed among the variables studied.

**Results:**

In a sample of 818 medical students, differences by school appeared in empathy, teamwork, lifelong learning, and family loneliness. In addition, family loneliness showed an inverse correlation with empathy, teamwork, and learning measures. While having a positive relationship with the mother was associated with a greater development of empathy and learning abilities in the entire sample, a similar effect was observed in father–son relationships, but only in the private medical school group. Finally, in the public medical group, a multiple regression model explained 43% of the variability of empathy based on a lineal relationship with teamwork (*p* < 0.001), lifelong learning (*p* < 0.001), and family loneliness (*p* < 0.001).

**Conclusion:**

These findings confirm how family loneliness is detrimental to the development of medical professionalism. Also, they support the important role that the family, and especially parents, plays in the development of empathy, teamwork, and abilities in medical students. Finally, these findings highlighted important differences among students enrolled in public and private medical schools.

## Introduction

### Parents as a Source of Social and Emotional Learning

Several authors agree that, due to their intensity, relationships between parents and their offspring have an important impact in adult offspring’s life course (Polenick et al., 2018). According to Fingerman (2001), the main reason is the complexity of these types of relationships: mostly long-standing and emotionally intense. In the case of the mother, this bond strengthens at the moment of the birth during skin-to-skin contact between mother and newborn baby (Hojat, 2016). Based on this, Bowlby (1982) proposed his “attachment theory” as an explanation for the dynamics (and its consequences) that accompanies the infant’s repeated encounters with primary caregivers. According to him, individuals develop internalized working models of their relationships as they move from childhood to adulthood. For some authors (Hojat, 2016), these early encounters, when they are positive, create a solid basement for later interpersonal and social relationships Ainsworth (1985a, b) argued, for example, that this type of attachment developed in early childhood is likely to endure throughout life. In adults, it has been demonstrated that securely attached children, who developed a sense of trust with their caregivers, develop the capacity to respond sensitively and empathically toward others in their social relationships (Kestenbaum et al., 1989). On the contrary, persons lacking a secure attachment with their mother during their childhood tend to express more aggressive, non-compliant, or, at least, less empathic behaviors in their adulthood (Hojat, 1998). In students of medicine, it has been reported that those who had a secure attachment history were more likely to choose specialties that require more interaction with patients (Ciechanowski et al., 2004; Hojat et al., 2005).

From another scope, the “intergenerational solidarity theory” states that positive and negative aspects of the current relationships with parents contribute to their adult offspring’s wellness and prosocial behaviors (Bengtson et al., 2002). Different studies provide research evidence supporting this theory. For example, it has been demonstrated that rewarding and supportive ties with parents are linked to better wellness, whereas strain or tension in parent–offspring relationships is associated with poor wellness in their adulthood (Fingerman et al., 2008). In a recent study in university students (Fingerman et al., 2012), researchers observed that parents and offspring are more involved in one another’s lives nowadays than in the past. This involvement, measured in the weekly frequency of phone conversations and in the frequency of parental financial, practical, and emotional support, was characterized as a contributor in students’ life satisfaction.

### Family Environment and Its Possible Influence in the Development of Empathy, Teamwork, and Lifelong Learning Abilities

Medical professionalism refers to the sets of skills and values that characterize the essence of humanism in physicians, regardless of geographical, social, or cultural differences (Vivanco and Delgado-Bolton, 2015). For measurement purposes, Veloski and Hojat (2006) suggest that medical professionalism is achieved by mastering three professional competences: empathy with patients, abilities toward inter-professional collaborative work with other healthcare professionals, and physician’s lifelong learning abilities. The influence of individual and environmental factors in the acquisition and development of these three competences has been studied extensively in the last years. Individual factors have been mainly associated with attitudinal abilities (Hojat and Zuckerman, 2008; O’Tuathaigh et al., 2019), while the environment has usually been associated with certain academic and clinical settings where professional training of medical students and physicians-in-training is performed (Hafferty and O’Donnell, 2015; San-Martín et al., 2016). In fact, a hierarchical environment has been associated with a poor development of teamwork abilities in medicine in comparison with nursing (Hojat et al., 1997, 2003; San-Martín et al., 2017; Tuirán-Gutiérrez et al., 2019; Berduzco-Torres et al., 2020). On the contrary, facilitating environments, either academic (Smith et al., 2016) or professional (Bernabeo et al., 2018), have shown a positive effect on the development of medical professionalism. In addition, this experimental evidence suggests that, in earlier stages of personal and professional development, individuals are more sensitive to the influence of their closest environment. Then, it is expected that family, the first and most meaningful environment where individuals acquire self-esteem, self-motivation, and social skills, can play an important role of influence in the development of certain abilities associated with professional competences, such as empathy, teamwork, or lifelong learning abilities. In the last 30 years, increasing empirical evidence reported from different cultural settings has supported the role of influence that the family environment possibly plays in medical professionalism. For example, having family problems was associated with a decreased academic performance and an increased attrition rate in medical students (Biggs et al., 1991). Also, medical students who reported during their childhood high dissatisfaction with their parents (Hojat et al., 1990) or mostly unavailable mothers (Hojat, 1996) showed higher intensity and chronicity on loneliness and depression, low self-esteem and well-being, more difficulties to address difficult stress events, and less satisfaction with peer relationships. These findings acquire special relevance taking into consideration two facts that have been proven in recent studies with healthcare professionals: (i) an inverse association between loneliness and empathy (Marilaf-Caro et al., 2017; Soler-Gonzalez et al., 2017) and (ii) the inverse association between empathy and work distress (Marilaf-Caro et al., 2017; San-Martín et al., 2017; Yuguero et al., 2017). More recently, one study reported in Spain that nursing students with positive mother–son relationships showed less loneliness and slightly greater empathetic abilities with patients (Domínguez et al., 2017). Finally, in Chinese students of medicine (Li et al., 2018), it was reported that a positive parental rearing pattern helped in the cultivation of empathetic abilities in patient’s care. In societies similar to the Peruvian one, in which university students are often still living with their parents or close relatives, it is expected that the family environment plays an important role of influence in the development of students’ self-motivation and social skills.

Based on the aforementioned framework, this study was performed with the purpose of confirming the following hypothesis: In the absence of a specific training in empathy and teamwork and lifelong learning abilities, their development in medical students is associated with students’ loneliness perception in their family environment. Three research objectives were established: (i) to analyze the type of association existing between family environment and empathy, teamwork, and lifelong learning measures; (ii) to identify differences in empathy, teamwork, lifelong learning measures, and family environment according to the variables collected (sex, medical school, academic achievement, and students’ place of origin); and (iii) in those cases where differences were confirmed, to characterize the factors of influence in their development in separate groups.

## Materials and Methods

### Participants

As inclusion criteria, this study included a sample composed only of undergraduate students matriculated in the only two medical schools, one private and the other one public, existing in Cusco City (Peru). In the academic year 2018–2019, when this study was performed, the number of undergraduate students matriculated in both medical schools was 1,410. Taking this into consideration, the sample size estimated for this study was 784 students (for a confidence level of 95% and a precision of 3.5%).

According to the medical curricula developed by both institutions, those students were distributed into two groups: students enrolled in preclinical undergraduate programs (from the first until the third academic year) and students enrolled in clinical undergraduate programs (from the fourth until the sixth academic year). Neither of the two medical schools offers training courses specifically oriented toward the development of empathy, inter-professional collaborative work (teamwork), or lifelong learning in its medical curricula.

For the exclusion criteria: undergraduate students of other disciplines different from medicine and undergraduate medical students who were attending communitarian work in isolated rural communities, and those who were attending internship programs in institutions out of Cusco, were excluded from this study.

### Empathy, Teamwork, and Lifelong Learning Measures

Empathy was measured with the medical student version of the Jefferson Scale of Empathy (JSE-S) (Hojat et al., 2001). The JSE-S evaluates students’ orientation toward empathetic relationships with patients. The scale is composed of 20 items that are answered following a Likert scale from 1 (*strongly disagree*) to 7 (*strongly agree*). A higher score means that the student has a greater orientation or behavioral tendency toward empathic engagement in patient care (Hojat, 2016).

Teamwork was measured with the Jefferson Scale of Attitudes toward Physician–Nurse Collaboration (JSAPNC) (Hojat et al., 1999). The JSAPNC was originally developed to address areas of physician–nurse interaction, including authority, autonomy, shared responsibilities in patient care, collaborative decision making, and role expectations (Hojat et al., 1999). The scale is composed of 15 items that are answered following a Likert scale from 1 (*strongly disagree*) to 4 (*strongly agree*).

Finally, lifelong learning was measured with the medical student version of the Jefferson Scale of Physicians Lifelong Learning (JeffSPLL-MS) (Wetzel et al., 2010). The JeffSPLL-MS measures the development of skills related to information gathering, the use of learning opportunities, and self-motivation. The scale is composed of 14 items that are answered following a Likert scale from 1 (*strongly disagree*) to 4 (*strongly agree*).

### Family Environment Measures

The father–son and mother–son relationships, and students’ perception of loneliness in their family, were used as measures of family environment. Students’ perception of their relationship with the mother was measured using the following statement: “the relationship with my mother is.” Respondents were invited to answer using a Likert scale from 1 (*There is no relationship*) to 8 (*Excellent*). Perception of the relationship with the father was calculated in a different item by replacing mother with father in the aforementioned statement. Family loneliness was measured using the family domain of the short version of the Social and Emotional Loneliness Scale for Adults (SELSA-S) (Ditommaso et al., 2004). The SELSA-S produces a global loneliness score based in 15 items, as well as scores for three domains of loneliness: family, romantic, and social. Higher scores in the scale indicate a higher perception of loneliness. The family domain of the SELSA-S is composed of five items that are answered following a Likert scale from 1 (*strongly disagree*) to 7 (*strongly agree*).

### Sociodemographic Information

Finally, sex, age, medical school (public or private medical school), academic achievements (students enrolled in the preclinical or clinical phases of the undergraduate medical studies), and students’ place of origin (originally from Cusco City or not) were collected to determine possible biases in the development of the elements measured.

### Procedures

The aforementioned measures were included in a unique questionnaire. This questionnaire was administered to undergraduate medical students in the two participating institutions. Questionnaires consisted of paper forms provided together with a pencil and an information letter in enclosed envelopes. Once questionnaires were responded to, they were returned in their envelopes to local researchers following a general protocol previously approved by the Comité Ético de Investigación de La Rioja, an independent ethics committee (ref. CEICLAR-PI-199), in November 2016. After obtaining the administrative authorization from the two institutions participating in this study (March 2018), both medical schools provided administrative support to the process of distribution and collection of questionnaires. The participation of undergraduate medical students was voluntary, anonymous, and secret. The process was carried out in accordance with the ethical principles for medical research involving human subjects of the Declaration of Helsinki. There was no potential risk for the participants, and anonymity was guaranteed throughout the entire data collection and later analysis.

### Data Analysis

In the analysis, only the scales with all the items answered were included and those in which the Cronbach’s alpha coefficient was higher than 0.70.

In the entire sample, a multiple regression analysis was performed to determine the factors of influence on the three elements measured for medical professionalism. However, the regression models obtained did not comply with all the conditions that are necessary for statistical inference. Therefore, in the entire sample, an analysis of variance (ANOVA) was used to compare empathy, teamwork, lifelong learning, and family environment measures according to sex, medical school, academic achievements, and place of origin in order to determine differences by study groups. In these analyses, partial eta-squared values were calculated to estimate the size effect of each variable, while the others with statistical difference were controlled. Effect sizes less than 0.01 were considered negligible, thus practically insignificant (Hojat and Xu, 2004; Pierce et al., 2004; Lakens, 2013). On the contrary, effect sizes between 0.01 and 0.058 were interpreted as small, between 0.059 and 0.137 were considered as medium, and equal to or greater than 0.138 were interpreted as large (Lawson et al., 2019). As normality was not achieved, Spearman’s correlation coefficients were used to calculate the associations between empathy, teamwork, and lifelong learning measures and family environment measures in the entire sample. The entire sample was divided into study groups in those cases where differences in empathy, teamwork, and lifelong learning measures were confirmed, and further analyses were performed comparing separate groups.

All analyses were done in R language, version 3.5.2, for Windows. In the statistical analysis, *nortest* (Gross and Ligges, 2015), *apaTABLE* (Stanley, 2018), and *multilevel* (Bliese, 2016) packages were included.

## Results

The participants included 818 students (349 males and 469 females), totaling 82% of all the undergraduate students enrolled in the two medical schools of Cusco. Of them, 238 belonged to the public medical school and 580 belonged to the private one. The average age was 21 years (*SD* = 3), with a range between 17 and 51 years. Based on their place of origin, 547 were originally from Cusco City and 271 were students coming from other cities. According to academic achievements, 617 students were enrolled in the preclinical phase of their medical studies and 201 in the clinical phase. All the scales used presented adequate psychometric properties with alpha coefficient properties over 0.8 (see Table 1).

**TABLE 1:** Descriptive analysis of the measures of empathy, teamwork, lifelong learning, and family loneliness.

**Statistics**	**JSE-S**	**JSAPNC**	**JeffSPLL-MS**	**SELSA-S/FD**
*n*	790	801	808	808
**Global scores**				
Possible range	20–140	15–60	14–56	5–35
Observed range	30–140	18–60	14–56	5–35
Median	103	43	44	12
Mean	100	43	43	13
Standard deviation	19	7	7	7
Alpha coefficient	0.87	0.82	0.87	0.81

**JSE-S, medical students’ version of the Jefferson Scale of Empathy; JSPANC, Jefferson Scale of Attitudes toward Physician–Nurse Collaboration; JeffSPLL-MS, medical students’ version of the Jefferson Scale of Physicians Lifelong Learning; SELSA-S/FD, family domain of the Social and Emotional Loneliness Scale for Adults.**

### Analyses in the Entire Sample

A multiple regression analysis was performed in the entire sample in order to determine the factors of influence on the three elements measured for medical professionalism. However, the regression models obtained in this analysis did not comply with all the conditions that are necessary for statistical inference (normality, zero mean, constant variance and incorrectness of the residuals, and linearity and absence of multicollinearity).

Therefore, a correlation analysis was performed to determine the type of association existing between empathy, teamwork, and lifelong learning scores and family environment measures (first research objective). Correlation analyses confirmed a direct relationship between having a positive relationship with the mother and global scores of medical empathy (*ρ* = 0.15, *p* < 0.001) and lifelong learning (*ρ* = 0.13, *p* < 0.001). A similar association was also observed for the father–son relationship, but only in the case of lifelong learning (*ρ* = 0.11, *p* = 0.001). On the contrary, perceiving loneliness in the family showed a negative correlation with global scores of empathy (*ρ* = −0.54, *p* < 0.001), teamwork (*ρ* = −0.27, *p* < 0.001), and lifelong learning (*ρ* = −0.44, *p* < 0.001). A complete summary of this analysis is shown in Table 2.

**TABLE 2:** Spearman’s correlation coefficients for empathy, teamwork, and lifelong learning according to family environment measures.

	**Empathy**	**Teamwork**	**Lifelong learning**
**Family loneliness**			
Entire sample	−0.54***	−0.27***	−0.44***
Private medical school group	−0.55***	−0.27***	−0.45***
Public medical school group	−0.51***	−0.20**	−0.39***
**Positive mother–son relationship**			
Entire sample	0.15***	0.02	0.13***
Private medical school group	0.17***	0.03	0.13**
Public medical school group	0.19**	0.01	0.17**
**Positive father–son relationship**			
Entire sample	0.05	−0.04	0.11**
Private medical school group	0.08	−0.01	0.13**
Public medical school group	0.05	0.08	0.09

**p < 0.01; ***p < 0.001.

### Analyses in Separate Groups

Based on the outcomes observed in previous analyses, an analysis of variance was performed for each main measure studied in order to identify differences that were associated with some of the variables collected in the sociodemographic form (second research objective).

**FIGURE 1:**
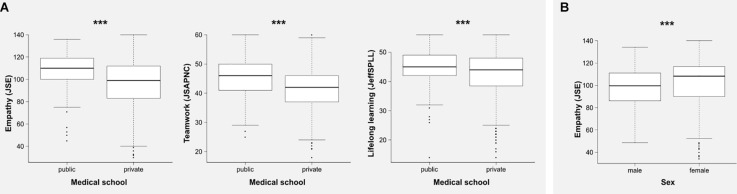
Comparisons of the global scores of empathy (Jefferson Scale of Empathy, JSE), teamwork (Jefferson Scale of Attitudes toward Physician–Nurse Collaboration, JSPANC), and lifelong learning (Jefferson Scale of Physicians Lifelong Learning, JeffSPLL) according to medical school **(A)** and sex **(B)**. ***p < 0.001.

A two-way ANOVA, in the case of empathy, confirmed differences according to the “sex” and “medical school” variables (Figure 1), with small (η*_p_*^2^ = 0.02) and medium (η*_p_*^2^ = 0.08) effect sizes, respectively. In the case of teamwork, a two-way ANOVA also confirmed differences according to the “medical school” (Figure 1) and “academic achievement” variables. However, while “medical school” showed a medium effect size (η*_p_*^2^ = 0.06), the effect size for “academic achievement” was negligible (η*_p_*^2^ = 0.009). Finally, a two-way ANOVA confirmed differences in global scores of lifelong learning according to “medical school” (Figure 1) and the interaction of “medical school by sex.” But only in the case of “medical school” was the effect size important (η*_p_*^2^ = 0.02). The summary of these findings is shown in Table 3.

**TABLE 3:** Two-way ANOVA of empathy, teamwork, and lifelong learning by medical school, sex, and academic achievement, as sources of variation.

**Source of variation**	**Empathy**			
	***F*_(1, 785)_**	**η^2^**	**η*_p_*^2^**	***p***
**Main effects**				
Medical school (public vs. private)	69.49	0.02	0.02	<0.001
Sex (male vs. female)	19.43	0.08	0.08	<0.001
**Two-way interaction**				
Medical school–sex	1.72	0.002	0.002	0.19

**Source of variation**	**Teamwork**			
	***F*_(1, 796)_**	**η^2^**	**η*_p_*^2^**	***p***

**Main effects**				
Medical school (public vs. private)	48.99	0.06	0.06	<0.001
Academic achievement (pre-clinics vs. clinics)	7.42	0.009	0.009	0.007
**Two-way interaction**				
Medical school–academic achievement	0.47	0.00	0.00	0.49

**Source of variation**	**Lifelong learning**			
	***F*_(1, 802)_**	**η^2^**	**η*_p_*^2^**	***p***

**Main effects**				
Medical school (public vs. private)	20.58	0.02	0.02	<0.001
Sex (male vs. female)	2.64	0.003	0.003	0.10
**Two-way interaction**				
Medical school–sex	4.76	0.006	0.006	0.03

**F_(df)_, F-value_(degrees of freedom)_; η^2^, eta-squared; η_*p*_^2^, partial eta-squared; p, p-value.**

**FIGURE 2:**
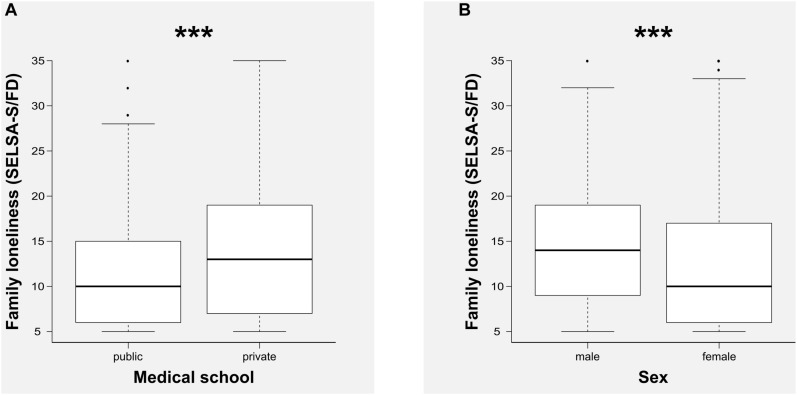
Comparisons of the scores of family loneliness (family domain of the Social and Emotional Loneliness Scale for Adults, SELSA-S/FD) according to medical school **(A)** and sex **(B)**. ^∗∗∗^*p* < 0.001.

A two-way ANOVA also confirmed that students’ perception of family loneliness was greater in male students (*p* < 0.001, η*_p_*^2^ = 0.03) and in students matriculated in the private medical school (*p* < 0.001, η*_p_*^2^ = 0.02), as is shown in Figure 2. On the other hand, two one-way ANOVA confirmed differences in mother–son relationships according to “sex” (*p* = 0.046) and in father–son relationships according to “medical school” (*p* = 0.009). However, in both cases, analyses revealed that the effect sizes were negligible (η*_p_*^2^ < 0.01). A summary of these findings is presented in Table 4.

**TABLE 4:** ANOVA of family loneliness and mother–son and father–son relationships by medical school and sex, as sources of variation.

**Source of variation**	**Family loneliness**			
	***F*_(1_, _802)_**	**η^2^**	**η*_p_*^2^**	***p***
**Main effects**				
Medical school (public vs. private)	19.70	0.02	0.02	<0.001
Sex (male vs. female)	23.36	0.03	0.03	<0.001
**Two-way interaction**				
Medical school–sex	2.70	0.003	0.003	0.10

**Source of variation**	**Mother–son relationship**			
	***F*_(1_, _813)_**	**η^2^**	**η*_p_*^2^**	***p***

**Main effect**				
Sex (male vs. female)	4.00	0.005	0.005	0.046

**Source of variation**	**Father–son relationship**			
	***F*_(1_, _813)_**	**η^2^**	**η*_p_*^2^**	***p***

**Main effect**				
Medical school (public vs. private)	6.91	0.008	0.008	0.009

**F_(df)_, F-value_(degrees of freedom)_; η^2^, eta-squared; η_*p*_^2^, partial eta-squared; p, p-value.**

Separate correlation analyses performed in both medical school groups showed associations similar to the ones reported in the entire sample, with the exception of the father–son relationship. Indeed, having a positive relationship with the father showed a positive correlation with empathy only in the case of students matriculated in the private university (*ρ* = 0.13, *p* = 0.001). In students enrolled in the public university, this association was not confirmed (*ρ* = 0.09, *p* = 0.18), as is shown in Table 2. Separate correlation analyses by sex groups showed similar associations between empathy and family environment measures to the ones reported in the entire sample. Similar findings were also observed for family environment measures and teamwork abilities in the correlation analyses done in student groups according to academic achievements.

Based on the results observed in the aforementioned analyses, a multiple lineal regression analysis was performed separately in both medical school groups (third research objective). From this analysis, a regression model explaining the 43% variance in empathy was obtained, but only for the public medical school group (Table 5). According to this model, teamwork (*p* < 0.001), lifelong learning (*p* < 0.001), and family loneliness (*p* < 0.001) appeared as influencing factors in the development of medical empathy. This model complied with all the conditions necessary for statistic inference.

**TABLE 5:** Multiple regression model for empathy global scores of undergraduate students matriculated in the public medical school of Cusco.

**Predictors**	**β**	**SE**	***t***	***p***
Teamwork abilities (JSAPNC)	+0.69	0.14	+4.89	<0.001
Lifelong learning abilities (JeffSPLL-MS)	+0.69	0.16	+4.30	<0.001
Family loneliness (SELSA-S/FD)	−0.84	0.15	−5.75	<0.001

**JSPANC, Jefferson Scale of Attitudes toward Physician–Nurse Collaboration; JeffSPLL-MS, medical students’ version of the Jefferson Scale of Physicians Lifelong Learning; SELSA-S/FD, family domain of the Social and Emotional Loneliness Scale for Adults; β, beta coefficient; SE, standard error; t, t experimental; p, p-value.**

## Discussion

The relationship between family environment and empathy, teamwork, and lifelong learning measures was confirmed in the entire sample and in separate groups. In the case of family loneliness, the inverse correlation with the three measured elements observed in this study is in consonance with findings reported in previous studies performed in Latin American and Spanish healthcare professionals (Marilaf-Caro et al., 2017; Soler-Gonzalez et al., 2017) and in Spanish nursing students (Domínguez et al., 2017). Regarding the figure of the mother and the father, the findings observed in this study suggest that the mother plays a positive role of influence in the development of empathy and learning abilities. This role has been previously described in students of medicine (Hojat et al., 2005) and nursing (Domínguez et al., 2017), but only in relation to empathy. Furthermore, the role that the mother (in the entire sample) and the father (in the private medical school group) have in the improvement of learning abilities is quite an innovative finding this study is reporting as, up to now, the available studies in the field had described this effect only in children and teenagers, but not in adults. In fact, in teenagers, the mother (De la Iglesia et al., 2014) and the father (Cooper, 2009) have been described as the main sources of psychological and motivational support with an important effect in the academic success of their children.

The findings observed in this study reveal important differences in the elements measured according to medical school (private vs. public). Students matriculated in the private university presented lower scores on empathy, teamwork, and lifelong learning in comparison to those enrolled in the public one. In addition, students enrolled in the public medical school presented better indicators on family environment measures. Taking into consideration that the differences reported in the elements measured for medical professionalism are not associated with the medical program of both institutions (neither of the two medical schools participating in this study offers training courses specifically oriented toward empathy, teamwork, or lifelong learning in their formal curricula), a plausible explanation for this difference is the recognized social and economic inequalities existing in Peru and in other Latin American countries. The findings showed that students matriculated in the private medical school perceived more family loneliness than those matriculated in the public one. These findings are in consonance with other studies where it has been described that caregivers’ educational history and personal experiences play an important role of influence in the professional aspirations of Peruvian young adults (Benavides et al., 2006; Crivello, 2011; Mena, 2012; Guerrero et al., 2016). In Peruvian families, especially in the rural areas, reaching higher education has a great value as a way to “become somebody” (Crivello, 2011) and an escape from poverty (Boyden, 2013). In private institutions, where students must pay a fee, studying medicine implies an important investment for families. In consequence, parents tend to spend more time at work and less time with their children. It is reasonable that, under these circumstances, medical students perceive more family loneliness, acquire more commitment toward their caregivers, but also are under more stress caused by the fact of being an economic burden for their families. On the other hand, in family groups with parents more dedicated to work instead of spending time with their family, it is possible that children tend to develop less social abilities than those who spend more time with their parents. In accordance with this, some studies highlighted some significant differences in working opportunities (Hernández-Gálvez and Roldán-Valadez, 2019) and in the election of specialty (Gutiérrez-Cirlos et al., 2017) in medical students associated with the medical school, private or public, where they studied their career. In a recent study, Li and colleagues reported that medical students from the best-ranked American universities tend to develop less altruistic and humanitarian professional attitudes (Li et al., 2017). In accordance with this, the lower development of empathy, teamwork, and lifelong learning abilities among students enrolled in the private medical school of Cusco can be another consequence of the exposition to a more elitist professional environment in the absence of a targeted educational program on these three abilities.

Differences in empathy and in family environment also appeared according to sex. These differences, in the case of empathy, are in accordance with the gender differences reported in social behavior, interpersonal abilities, and empathetic engagement (Hojat, 2016). In the specific case of medical empathy, similar differences have been described in studies with undergraduate medical students in the United States (Hojat et al., 2002, 2005), Spain, and Latin American countries (Alcorta-Garza et al., 2016). Social learning, role expectations, sociocultural factors, evolutionary history, and hormonal and physiological differences have been described as some plausible causes of such differences in empathetic abilities (Hojat, 2016). The findings also revealed that male students perceive more family loneliness than do their female peers. These findings are also consistent with some characteristics and parenting behavior styles that are still dominant in Peruvian society, which are associated with family kinship, household structures, and social stereotypes related to gender roles (Scott, 1990; Aufseeser, 2019).

When medical school groups were analyzed separately, a regression model in the public medical school group revealed that teamwork and lifelong learning play a role of positive influence in the development of empathy. This finding brings new evidence supporting the role of these three elements as specific components of medical professionalism (Veloski and Hojat, 2006). In addition, this analysis confirmed the negative influence that family loneliness has in the development of medical empathy in those students. The impossibility of obtaining a regression model in the private medical school group reinforces the idea that there are many more factors of influence in this group, probably associated with some other aspects that have not been covered in this study and could be associated with individual and environmental factors.

## Conclusion

In conclusion, these findings confirm that the early development of empathy, teamwork, and lifelong learning abilities, as part of their professional medical competences, is sensitive to the influence of factors related to the family environment, especially in the absence of targeted educational programs included in the medical curricula. These findings also bring new evidence supporting the important role that family plays in the development of professionalism in undergraduate medical students.

### Limitations

This study is mainly aimed at the specific situation of students during their medical degree. Therefore, these findings are not necessarily transferable to postgraduate education or professional work. Further studies are also needed to characterize the factors influencing the development of medical professionalism in students matriculated in private medical schools of Peru since it seems that the family environments of the students matriculated in these institutions present some important differences in comparison to those who are in public schools. Furthermore, this study was performed in the only two medical schools located in one Peruvian city in the Andean region. Even though Cusco has a relevant importance in the Andean region of Latin America due to its strategic position and multicultural and multilingual social structure, further studies in other geographical and cultural contexts are required in order to determine the existence of possible similarities.

## Data Availability Statement

The raw data supporting the conclusions of this article will be made available by the authors, without undue reservation, to any qualified researcher.

## Ethics Statement

The studies involving human participants were reviewed and approved by the Comité Ético de Investigación de La Rioja (CEImLAR), Ref. CEICLAR-PI-199. The patients/participants provided their written informed consent to participate in this study.

## Author Contributions

LV was in charge of the study’s overall design and drafting of the manuscript. MS-M and LV performed the statistical processing of data. NB-T was in charge of the coordination with the participating institutions. BC-C and PM were in charge of the survey design, distribution, and data collection. LV and RD prepared the manuscript drafts. All authors contributed to the presented work, participated during the interpretation process of the results, and approved the final manuscript.

## Conflict of Interest

The authors declare that the research was conducted in the absence of any commercial or financial relationships that could be construed as a potential conflict of interest.

## References

[B1] Ainsworth M. D. (1985a). Attachments across the life span. *Bull. N. Y. Acad. Med* 61 792–812. 3864511 PMC1911889

[B2] Ainsworth M. D. (1985b). Patterns of infant-mother attachments: antecedents and effects on development. *Bull. N. Y. Acad. Med.* 61 771–791. 3864510 PMC1911899

[B3] Alcorta-Garza A. San-Martín M. Delgado R. Soler J. Roig H. Vivanco L. (2016). Cross-validation of the Spanish HP-version of the jefferson scale of empathy confirmed with some cross-cultural differences. *Front. Psychol.* 7:1002. 10.3389/fpsyg.2016.01002 27462282 PMC4940391

[B4] Aufseeser D. (2019). Mothering in the context of poverty: disciplining peruvian mothers through children’s rights. *Feminist Encount.* 3 13 10.20897/femenc/5919

[B5] Benavides M. Olivera I. Mena M. (2006). “De papás y mamás a hijos e hijas: las aspiraciones sobre el futuro yrol de las familias en las actividades escolares en el Perú rural,” in *Los desafíos de la Escolaridad en el Perú: Estudios Sobre Los Procesos Pedagógicos, Los Saberes Previos y el rol de las Familias*, ed. Benavides M. (Lima: GRADE), 157–214.

[B6] Bengtson V. Giarrusso R. Mabry J. B. Silverstein M. (2002). Solidarity, conflict, and ambivalence: complementary or competing perspectives on intergenerational relationships? *J. Marriage Fam.* 64 568–576. 10.1111/j.1741-3737.2002.00568.x

[B7] Berduzco-Torres N. Choquenaira B. Medina P. Chihuantito L. A. Caballero S. Gallegos E. (2020). Factors related to the differential development of Inter-professional collaboration abilities in medicine and nursing students. *Front. Psychol.* 11:432. 10.3389/fpsyg.2020.00432 32292364 PMC7135885

[B8] Bernabeo E. C. Chesluk B. Lynn L. (2018). Tiny moments matter: promoting professionalism in everyday practice. *J. Continuing Educ. Health Profess.* 38 110–116. 10.1097/CEH.0000000000000202 29782368

[B9] Biggs J. S. G. Najman J. M. Schulz E. B. Williams G. (1991). Parental problems influencing the academic achievement of medical students: a prospective study. *Med. Educ.* 25 374–382. 10.1111/j.1365-2923.1991.tb00084.x 1758313

[B10] Bliese P. (2016). *Multilevel: Multilevel Functions. R package version 2.6. The Comprehensive R Archive Network.* Available online at: https://CRAN.R-project.org/package=multilevel (accessed date June 20, 2020).

[B11] Bowlby J. (1982). *Attachment and loss. Vol. 1. Attachment.* New York, NY: Basic Books.

[B12] Boyden J. (2013). We’re not going to suffer like this in the mud: educational aspirations, social mobility and independent child migration among populations living in poverty. *Compare* 43 580–600. 10.1080/03057925.2013.821317

[B13] Ciechanowski P. S. Russo J. E. Katon W. J. Walker E. A. (2004). Attachment theory in health care: the influence of relationship style on medical students’ specialty choice. *Med. Educ.* 38 262–270. 10.1046/j.1365-2923.2004.01767.x 14996335

[B14] Cooper S. M. (2009). Associations between father-daughter relationship quality and the academic engagement of African American adolescent girls: self-esteem as a mediator? *J. Black Psychol.* 35 495–516. 10.1177/0095798409339185

[B15] Crivello G. (2011). Becoming somebody: youth transitions through education and migration in peru. *J. Youth Stud.* 14 395–411. 10.1080/13676261.2010.538043

[B16] De la Iglesia G. Hoffmann A. F. Liporace M. F. (2014). Perceived parenting and social support: can they predict academic achievement in Argentinean college students? *Psychol. Res. Behav. Manag.* 7 251–259. 10.2147/PRBM.S68566 25258563 PMC4172105

[B17] Ditommaso E. Brannen C. Best L. A. (2004). Measurement and validity characteristics of the short version of the social and emotional loneliness scale for adults. *Educ. Psychol. Measur.* 64 99–119. 10.1177/0013164403258450

[B18] Domínguez V. San-Martín M. Vivanco L. (2017). Relaciones familiares, soledad y empatía en el cuidado del paciente en estudiantes de enfermería. *Aten. Primaria* 49 56–57. 10.1016/j.aprim.2016.03.007 27350409 PMC6876022

[B19] Fingerman K. L. (2001). *Aging Mothers and Their Adult Daughters: A Study in Mixed Emotions.* New York, NY: Springer Publishing Company.

[B20] Fingerman K. L. Cheng Y. P. Tighe L. Birditt K. S. Zarit S. (2012). “Relationships between young adults and their parents,” in *Early Adulthood in a Family Context. National Symposium on Family*, Vol. 2 eds Booth A. Brown S. Landale N. Manning W. McHale S. (New York, NY: Springer), 59–85. 10.1007/978-1-4614-1436-0_5

[B21] Fingerman K. L. Pitzer L. Lefkowitz E. S. Birditt K. S. Mroczek D. (2008). Ambivalent relationship qualities between adults and their parents: implications for the well-being of both parties. *J. Gerontol. Ser. B* 63 362–371. 10.1093/geronb/63.6.P362 19092039 PMC2749877

[B22] Gross J. Ligges U. (2015). *nortest: Tests for Normality. R package version 1.0-4. The Comprehensive R Archive Network.* Available online at: https://CRAN.R-project.org/package=nortest (accessed June 20, 2020).

[B23] Guerrero G. Sugimaru C. Cussianovich A. De Fraine B. Cueto S. (2016). *Education Aspirations Among Young People in Peru and Their Perceptions of Barriers to Higher Education.* Oxford, UK: Young Lives.

[B24] Gutiérrez-Cirlos C. Naveja J. Sánchez-Mendiola M. (2017). Factores relacionados con la elección de una especialidad en medicina. *Invest. Educ. Méd.* 6 206–214. 10.1016/j.riem.2017.05.005 29414975

[B25] Hafferty F. W. O’Donnell J. F. (eds) (2015). *The Hidden Curriculum in Health Professional Education.* Hanover: Dartmouth College Press.

[B26] Hernández-Gálvez D. C. Roldán-Valadez E. (2019). Mexican ENARM: performance comparison of public vs. private medical schools, geographic and socioeconomic regions. *Salud Públ. México* 61 637–647. 10.21149/10078 31661741

[B27] Hojat M. (1996). Perception of maternal availability in childhood and selected psychosocial characteristics in adulthood. *Genet. Soc. Gen. Psychol. Monogr.* 122 425–450. 8976598

[B28] Hojat M. (1998). Satisfaction with early relationships with parents and psychosocial attributes in adulthood: which parent contributes more? *J. Genet. Psychol.* 159 203–220. 10.1080/00221329809596146 9595703

[B29] Hojat M. (2016). *Empathy in Health Professions Education and Patient Care.* Dordrecht: Springer.

[B30] Hojat M. Borenstein B. D. Shapurian R. (1990). Perception of childhood dissatisfaction with parents and selected personality traits in adulthood. *J. Gen. Psychol.* 117 241–253. 2212998

[B31] Hojat M. Fields S. K. Rattner S. L. Griffiths M. Cohen M. J. Plumb J. D. (1997). Attitudes toward physician-nurse alliance: comparisons of medical and nursing students. *Acad. Med.* 72 S1–S3. 10.1097/00001888-199710001-00001 9347721

[B32] Hojat M. Fields S. K. Veloski J. J. Griffiths M. Cohen M. J. Plumb J. D. (1999). Psychometric properties of an attitude scale measuring physician-nurse collaboration. *Eval. Health Profess.* 22 208–220. 10.1177/01632789922034275 10557856

[B33] Hojat M. Gonnella J. S. Nasca T. J. Fields S. K. Cicchetti A. Scalzo A. L. (2003). Comparisons of American, Israeli, Italian and Mexican physicians and nurses on the total and factor scores of the Jefferson scale of attitudes toward physician–nurse collaborative relationships. *Int. J. Nurs. Stud.* 40 427–435. 10.1016/S0020-7489(02)00108-6 12667519

[B34] Hojat M. Gonnella J. S. Nasca T. J. Mangione S. Vergare M. Magee M. (2002). Physician empathy: definition, components, measurement, and relationship to gender and specialty. *Am. J. Psychiatry* 159 1563–1569. 10.1176/appi.ajp.159.9.1563 12202278

[B35] Hojat M. Mangione S. Nasca T. J. Cohen M. J. M. Gonnella J. S. Erdmann J. B. (2001). The jefferson scale of physician empathy: development and preliminary psychometrics. *Educ. Psychol. Measur.* 61 349–365. 10.1177/00131640121971158

[B36] Hojat M. Xu G. (2004). A visitor’s guide to effect sizes–statistical significance versus practical (clinical) importance of research findings. *Adv. Health Sci. Educ.* 9 241–249. 10.1023/b:ahse.0000038173.00909.f6 15316274

[B37] Hojat M. Zuckerman M. (2008). Personality and specialty interest in medical students. *Med. Teach.* 30 400–406. 10.1080/01421590802043835 18569662

[B38] Hojat M. Zuckerman M. Magee M. Mangione S. Nasca T. Vergare M. (2005). Empathy in medical students as related to specialty interest, personality, and perceptions of mother and father. *Pers. Individ. Differ.* 39 1205–1215. 10.1016/j.paid.2005.04.007

[B39] Kestenbaum R. Farber E. A. Sroufe L. A. (1989). Individual differences in empathy among pre-schoolers: relation to attachment history. *New Direct. Child Dev.* 44 51–64. 10.1002/cd.23219894405 2771129

[B40] Lakens D. (2013). Calculating and reporting effect sizes to facilitate cumulative science: a practical primer for t-tests and ANOVAs. *Front. Psychol.* 4:863. 10.3389/fpsyg.2013.00863 24324449 PMC3840331

[B41] Lawson T. R. Faul A. C. Verbist A. N. (2019). *Research and Statistics for Social Workers. Bridging the Gap for a Better Tomorrow.* New York, NY: Routledge.

[B42] Li C. Q. Ma Q. Liu Y. Y. Jing K. J. (2018). Are parental rearing patterns and learning burnout correlated with empathy amongst undergraduate nursing students? *Int. J. Nurs. Sci.* 5 409–413. 10.1016/j.ijnss.2018.07.005 31406856 PMC6626275

[B43] Li J. Dow W. H. Kariv S. (2017). Social preferences of future physicians. *Proc. Natl. Acad. Sci. U.S.A.* 114 E10291–E10300. 10.1073/pnas.1705451114 29146826 PMC5715739

[B44] Marilaf-Caro M. San-Martín M. Delgado R. Vivanco L. (2017). Empathy, loneliness, burnout, and life satisfaction in Chilean nurses of palliative care and homecare services. *Enfermeria Clin.* 27 379–386. 10.1016/j.enfcle.2017.04.010 28587755

[B45] Mena M. (2012). (De) construyendo ilusiones. Cambios intergeneracionales y de género en las aspiraciones educativas y ocupacionales en las zonas rurales de Ayacucho. *Debates Sociol.* 37 5–42.

[B46] O’Tuathaigh C. M. Idris A. N. Duggan E. Costa P. Costa M. J. (2019). Medical students’ empathy and attitudes towards professionalism: relationship with personality, specialty preference and medical programme. *PLoS One* 14:e0215675. 10.1371/journal.pone.0215675 31048851 PMC6497245

[B47] Pierce C. A. Block R. A. Aguinis H. (2004). Cautionary note on reporting eta-squared values from multifactor ANOVA designs. *Educ. Psychol. Measur.* 64 916–924. 10.1177/0013164404264848

[B48] Polenick C. A. Fredman S. J. Birditt K. S. Zarit S. H. (2018). Relationship quality with parents: Implications for own and partner well-being in middle-aged couples. *Fam. Process.* 57 253–268. 10.1111/famp.12275 28004851 PMC5481501

[B49] San-Martín M. Delgado R. Vivanco L. (2017). Professionalism and occupational well-being: similarities and differences among Latin American health professionals. *Front. Psychol.* 8:63. 10.3389/fpsyg.2017.00063 28179893 PMC5263132

[B50] San-Martín M. Rivera E. M. Alcorta A. Vivanco L. (2016). Moral perception, educational environment, and development of medical professionalism in medical students during the clinical rotations in Peru. *Int. J. Ethics Educ.* 1 163–172. 10.1007/s40889-016-0017-8

[B51] Scott A. M. (1990). “Patterns of patriarchy in the peruvian working class,” in *Women, Employment and the Family in the International Division of Labour*, eds Stichter S. Parpart J. L. (London: Macmillan International Political Economy Series), 198–220. 10.1007/978-1-349-20514-1_8

[B52] Smith S. D. Dunham L. Dekhtyar M. Dinh A. Lanken P. N. Moynahan K. F. (2016). Medical student perceptions of the learning environment: learning communities are associated with a more positive learning environment in a multi-institutional medical school study. *Acad. Med.* 91 1263–1269. 10.1097/ACM.0000000000001214 27119332

[B53] Soler-Gonzalez J. San-Martín M. Delgado R. Vivanco L. (2017). Human connections and their roles in the occupational well-being of healthcare professionals: a study on loneliness and empathy. *Front. Psychol.* 8:1475. 10.3389/fpsyg.2017.01475 28900410 PMC5581877

[B54] Stanley D. (2018). *ApaTables: Create american psychological association (apa) style tables. R package version 2.0.5. The Comprehensive R Archive Network.* Available online at: https://cran.r-project.org/web/packages/apaTables/vignettes/apaTables.html (accessed June 20, 2020).

[B55] Tuirán-Gutiérrez G. J. San-Martín M. Delgado Bolton R. Bartolomé B. Vivanco L. (2019). Improvement of inter-professional collaborative work abilities in Mexican medical and nursing students: a longitudinal study. *Front. Psychol.* 10:5. 10.3389/fpsyg.2019.00005 30697172 PMC6340986

[B56] Veloski J. Hojat M. (2006). “Measuring specific elements of professionalism: empathy, Teamwork, and Lifelong learning,” in *Measuring medical professionalism*, ed. Stern D. (New York, NY: Oxford University Press), 117–146.

[B57] Vivanco L. Delgado-Bolton R. (2015). “Professionalism,” in *Encyclopedia of Global Bioethics*, ed. ten Have H. (Cham: Springer), 10.1007/978-3-319-05544-2

[B58] Wetzel A. P. Mazmanian P. E. Hojat M. Kreutzer K. O. Carrico R. J. Carr C. (2010). Measuring medical students’ orientation toward lifelong learning: a psychometric evaluation. *Acad. Med.* 85 S41–S44. 10.1097/ACM.0b013e3181ed1ae9 20881701

[B59] Yuguero O. Marsal J. Esquerda M. Vivanco L. Soler-Gonzalez J. (2017). Association between low empathy and high burnout among primary care physicians and nurses in Lleida, Spain. *Eur. J. Gen. Pract.* 23 4–10. 10.1080/13814788.2016.1233173 27723375 PMC5774288

